# Personal decision support for survivor engagement: formulation and feasibility evaluation of a conceptual framework for implementing online cancer survivorship care plans

**DOI:** 10.1186/s12911-020-1073-8

**Published:** 2020-03-23

**Authors:** Akshat Kapoor, Priya Nambisan

**Affiliations:** 1 Health Services and Information Management, East Carolina University, 600 Moye Blvd. (Mail Stop 668), Greenville, NC 27834 USA; 2 Department of Health Informatics and Administration, Social Media and Health Research & Training Lab, College of Health Sciences, University of Wisconsin – Milwaukee, Northwest Quadrant Building B, Rm #6410, 2025 East Newport Avenue, Milwaukee, WI 53201-0413 USA

**Keywords:** Cancer survivorship, Consumer health information, Decision-support systems, eHealth, Self-management

## Abstract

**Background:**

Although cancer survivorship care plans have been in use for several years, they have been shown to not be effective in meeting the long-term needs of cancer survivors, in addition being generic and passive in nature. Interactive survivorship care plans in the form of a personal decision support aid could provide an opportunity to not only engage survivors in their health care, but also capture meaningful treatment-related outcomes to use as a rich data source as the basis for making informed decisions. The objective of this research is to formulate an evidence-based model framework for implementing breast cancer survivorship guidelines via an online breast cancer survivorship care plan (SCP).

**Methods:**

The study was completed in three steps. In the first step, or the requirements gathering phase, we conducted personal interviews of breast cancer survivors to determine their use of the survivorship care plan (SCP) and related needs to determine core SCP functions and formulate an implementation framework for an online SCP. In the second step, we used the framework as a guide to design and develop the online SCP tool. Finally, in the third step, we conducted preliminary testing to determine the feasibility of the developed tool among online users.

**Results:**

Fifteen breast cancer survivors were consulted, who reported several issues from their use of the traditional paper-based SCP. Four themes were identified that represent the SCP’s core desired functions. Eight features were matched to implement these core functions. Using a personal decision approach, an online SCP tool called ACESO that incorporates these features and functions was developed. Preliminary feasibility testing yielded overall positive responses from breast cancer survivors (*n* = 51).

**Conclusion:**

Our study demonstrated that survivors face challenges from their use of a traditional paper-based SCP. The online SCP we developed is technically feasible and has the potential to effectively engage breast cancer survivors in self-management and shared decision-making with their clinicians and caregivers. Further testing is required to assess its usability and long-term impact.

## Background

With better than ever cancer treatments now available, the number of breast cancer (BC) survivors has grown exponentially in the past decades [1]. Intensive medications and therapies such as radiation, chemotherapy and hormonal therapy have led to BC survivors experiencing a wide spectrum of treatment-related side effects, including poor sleep quality [2–4], depression [5–7], anxiety [6, 8], impaired sexual function [9–11], weight gain [12–14] and fatigue [15, 16]. This is further compounded by the fact that several of these symptoms occur several weeks or months after having completed treatment. Therefore, it is crucial to provide adequate means to support cancer survivors in an active manner. This includes supportive resources for regular monitoring for recurrence (or metastasis), handling any related and non-related comorbidities, provide recommendations for preventive care as well as dealing with any long-term side effects from the treatment.

Patient-generated health data (PGHD) provides an opportunity to not only engage survivors in their health care, but also capture meaningful treatment-related outcomes to use as a rich data source as the basis for making informed decisions [17].

An integral component of survivorship care planning is the survivorship care plan (SCP), which is a document provided to each patient upon completing initial treatment [18]. This document summarizes the patient’s treatment history, including surgeries and medications, and provides an extensive list of all potential treatment-related side effects. This information contained in a conventional SCP is passive, usually in the form of a static paper document, and is subject to the survivors’ recall bias [19, 20]. Moreover, this standard discharge practice of providing conventional cancer survivorship plans has been shown to provide no additional benefit to survivors [21]. Therefore, alternative mechanisms for implementing SCPs to address these drawbacks, and improve their uptake warrant further investigation.

An extensive review of currently available survivorship tools and resources revealed other online survivorship care plan tools do exist [22–25], however they only provided templates, allowing survivors to create and build their own SCP once and either print or save it electronically. While these resources are valuable for survivors who did not receive an SCP from their health care provider, they do not offer any additional benefit over a static, paper-based SCPs besides being accessible online, and lacked any additional interactive features, such as self-monitoring and tracking. On the other hand, interactive communication systems have been shown to educate and inform breast cancer survivors with various aspects of life after breast cancer [26], thus developing an interactive breast cancer survivorship care plan could be very beneficial to BC survivors. Furthermore, owing to longer survivor lifespans, cancer survivorship today is considered in the context of a chronic health condition, thus requiring modification in care planning, as survivor care needs evolve in the long term. Subsequently, ACESO needed to track and support BC survivors over a long period and respond to their most current health care needs at any given time.

Guided by the Theory of Patient and Consumer Activation [27], we seek to develop a framework that engages the survivors in playing an active role in managing their own health, thereby improving their self-management skills, confidence, and knowledge of their health condition. Since survivors have significantly less interaction with clinicians after completing initial cancer treatment, patient activation acknowledges that patients are responsible for managing their own health condition in the vast majority of time in between clinic visits, and that patients routinely make decisions that impact their health condition [28].

The objective of this research is to answer the following research questions: 1) What are the issues, challenges and unmet needs arising from the survivors’ use of their SCP?; and 2) What is the technical feasibility of designing, developing, and implementing an online SCP that would address the unmet needs of survivors?

## Methods

**Fig. 1:**
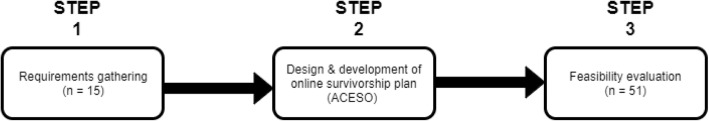
The three steps of the study *(created with draw.io v. 12.4.2)*

Using feedback from a cohort of BC survivors, we formulate an evidence-based model framework for implementing breast cancer survivorship guidelines. Guided by this framework, we developed and present a conceptual web-based BC survivorship and personal decision support tool called After Cancer Education and Support Operations (ACESO), which provides an interactive way for patients to manage their condition and deliver timely risk-adapted alerts based on information contained in their personalized SCP and the collected PGHD. Named after the Greek goddess of healing, ACESO aims to be an educational, personal decision support tool, which attempts to transform the current discharge process for breast cancer patients. The following sections describe the three steps performed as part of the study (Fig. 1).

### Step 1 – requirements gathering and formulation of conceptual framework

Participants for this step were recruited via posting flyers at prominent locations on the campus, and emails to volunteers at a local breast cancer resource center. To be considered eligible to participate in this step of the study, participants must a) have completed initial breast cancer treatment including surgery, radiation and/or chemotherapy; b) be currently cancer free; c) have no prior history of any other form of cancer, d) have received an SCP from their oncologist; e) have the ability to read and write in English; f) not be receiving treatment for any major disabling medical or psychiatric condition. After completion of initial screening, registration and obtaining informed consent, we conducted individual, face-to-face, open-ended interviews of 15 BC survivors who self-referred to participate in the study, in order to understand their use of their paper-based SCP, barriers to its use, and associated unmet needs (Additional file 1). The respondents’ SCP needs were used as a proxy for the desired core SCP functions to formulate the conceptual framework for the online SCP. All interviews were audio-taped and subsequently transcribed, checked for accuracy, and imported into NVivo (version 11). The qualitative data collected from the interviews was analyzed using thematic analysis [29]. We adopted a deductive approach for reviewing the transcribed interviews to identifying themes representing the survivors’ needs from the SCP. AK reviewed all transcripts through an iterative, open-coding process. In the unitizing stage, words or phrases within the transcribed text were tagged using codes describing the respondents’ challenges and unmet needs arising from use of their current SCP. Subsequently, semantic themes representative of these ideas were derived from the generated codes to generate the conceptual framework. This process was iterated after the completion of each interview, until data saturation was achieved, and no additional themes could be identified. To validate the results of the analysis, PN independently reviewed all coded transcripts for consistency and met periodically with AK to resolve any coding disagreements.

### Step 2 – design and development of ACESO

To maximize accessibility, and minimize the need for additional client resources, the system was designed as a web-based tool, requiring just a web-enabled device (personal computer, smartphone, tablet, etc.) with a browser, without the need to install any additional software. Utilizing the Web 2.0 list of participatory web features as a reference [30], we matched appropriate web-based features as solutions to the previously identified core SCP functions, in order to conceptualize the implementation framework. Web 2.0 features have been shown to be effective in engaging individuals managing chronic illnesses and training them to participate in shared decision making, and self-management of health conditions [31, 32]. Therefore, we adopted a personal decision support approach with the aim to further engage survivors and make their experience with the SCP more interactive with two-way communication. By incorporating survivor feedback to transform the conventional, paper-based SCP into a more personalized and interactive survivorship resource, ACESO enables two-way interaction with the SCP, by allowing patients to use the built-in tools to track survivor symptoms and quality of life observations, and in return, receive timely and customized risk-adapted alerts, and timely reminders for follow-up visits.

ACESO is supported by Windows Server 2016 for web hosting, PHP (PHP: Hypertext Preprocessor) for server-side scripting, and a MySQL Server database engine at the backend. After completing user registration using their conventional SCP to enter their BC related medical history as structured data, a personal account, secured by a user-specified username and password is created. A user may subsequently access their SCP by logging in via any web-enabled device connected to the Internet.

**Fig. 2:**
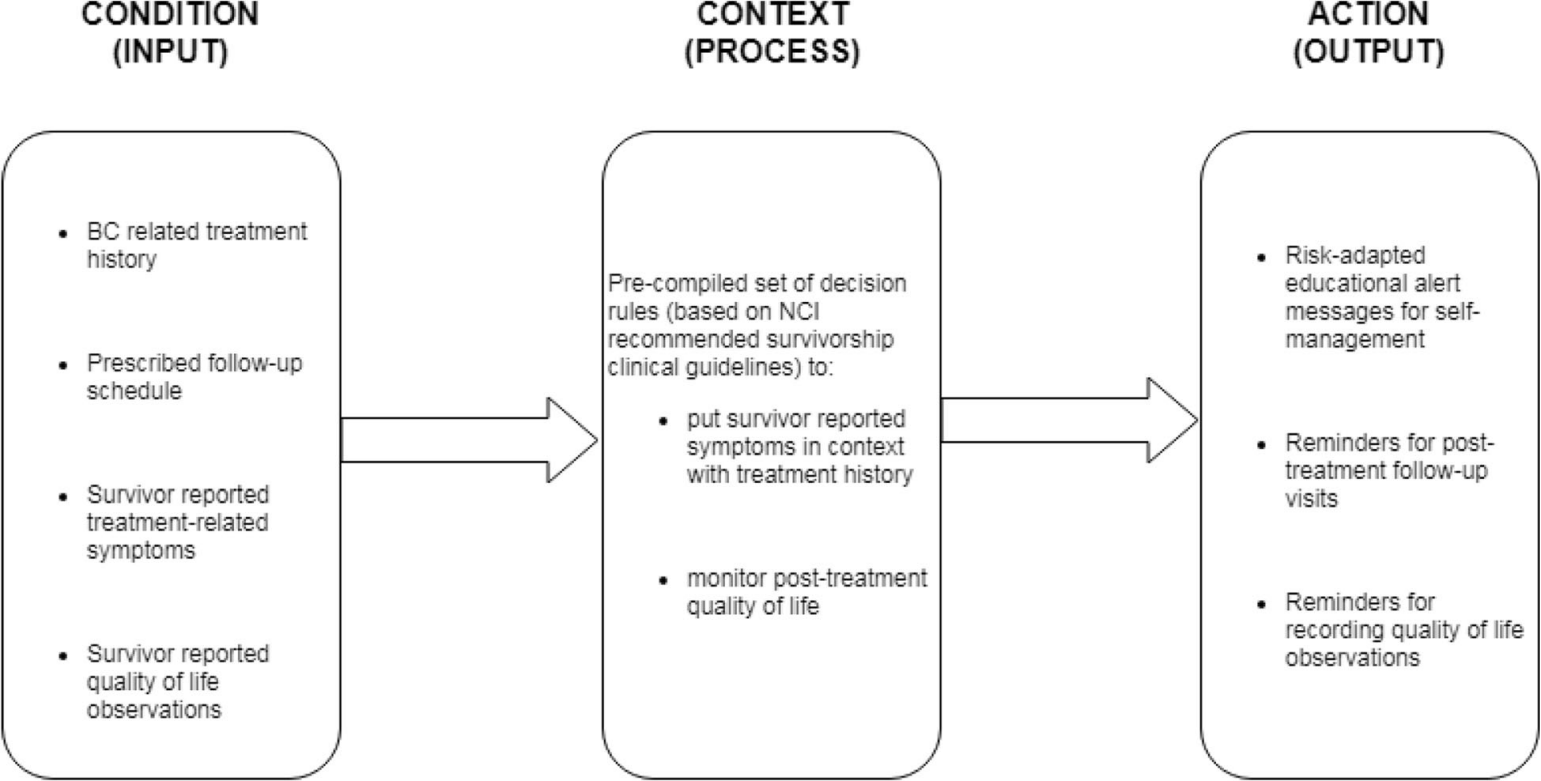
Conceptual functioning of the personal decision-support in ACESO, and its data sources *(created with draw.io v. 12.4.2)*

The decision rules to implement the personal decision support were derived from evidence-based BC survivorship care plan recommendations provided by the National Cancer Institute (NCI). Based upon clinical guidelines issued by the American Society for Clinical Oncology (ASCO), the NCI plan [33] contains comprehensive recommendations for follow-up visits, lists various cancer treatment-related side-effects, the corresponding populations at risk, and the recommended interventions to manage the side-effects. The knowledge contained in these guidelines is represented in the form of a set of pre-compiled decision rules that determine when any feedback, for example a reminder or an alert message should be pushed to the user. These rules are used to monitor and analyze the information collected from the patients, put them in context of their BC-related medical history, and push any warnings or alerts to the user, if needed. The conceptual model for the personal decision support, as well as corresponding data sources are described in Fig. 2.

All decision rules were validated for content accuracy and relevance by a BC oncologist and an oncology nurse practitioner. As a patient managed tool, ACESO is designed for use by all BC survivors, irrespective of their health care provider. We utilize the SNOMED-CT [34], standardized terminology to implement the decision support and describe various clinical terms such as diagnoses, symptoms, procedures, and medications used by ACESO.

### Step 3 – feasibility evaluation

We recruited BC survivors via email, flyers and postings on cancer survivorship communities on social media, to help us perform initial feasibility testing of the online BC survivorship tool. To be included in the study, survivors must have (i) completed initial treatment of their BC (radiation, chemotherapy, and/or surgery); (ii) have no prior history of any other cancers; and (iii) the ability to read and understand 8th grade level English. Survivors self-referred to participate and informed consent was obtained prior to participant registration. Six weeks after registration, we invited each user to complete a web-administered survey, to allow them adequate time to use the tool and provide preliminary feedback. A $50 Amazon gift card was offered to participants upon completing all study activities: registering their account; using ACESO over a period of 6 weeks; and completing the feedback survey, as reimbursement for their time spent to participate in the study.

## Results

### Step 1 – requirements gathering and formulation of conceptual framework

Survivors reported several barriers and unmet needs associated with the use of their conventional SCP. Based on analysis of feedback from the survivors’, four high level themes representing survivors’ SCP needs were identified: 1) education; 2) self-management; 3) adherence to recommended follow-up; and 4) social support (Table 1).

**Table 1:** Semantic themes regarding SCP related needs identified in requirements analysis phase

Themes	Examples of respondents’ feedback
**Education**	*“I often go to WebMD to do my own research, but I find it hard to understand if what I’m reading applies to me or not.”*
**Self-management**	*“It was so frustrating. After cancer I would get tired so easily, even when doing simple chores. Then someone suggested yoga which helped a lot”.*
**Adherence**	*“It hasn’t been easy since I got home. I’m trying to catch up with everything I missed at home and work, on top of keeping track of when I have to go back (for follow-up)”.*
**Support**	*“I personally belong to (name of local breast cancer resource center omitted for privacy), so now I have a mentor who is also a survivor and has been through it all. It’s nice knowing I’m not alone and have someone I can talk to”.*

A primary concern was a lack of understanding of the cause of specific treatment-related side effects, and unpreparedness to handle them. It was challenging for survivors to determine if the symptoms they were experiencing were normal, or to identify specifically which of all the exhaustive recommendations listed in the SCP were relevant to them.

The need for self-management of treatment-related symptoms was also prevalent in the respondents’ interviews. Survivors reported that while their SCPs contained an exhaustive list of common treatment-related symptoms, they do not always provide any accompanying information on the self-management of these symptoms. With limited access to clinicians post cancer treatment, survivors often felt unprepared in managing long-term treatment-related side effects at home.

Barriers to keeping up with SCP recommended follow-up was a recurring theme in the survivors’ narratives. Survivors are recommended several follow-up visits, including post-treatment mammography, physical check-ups, bone densitometry. These visits often recur at varying intervals, based on the type of follow-up, and time since completing treatment, making it challenging for survivors to stay current with, and plan upcoming follow-up appointments.

Another concern noted by survivors was the lack of available support resources that provided continued support after completing cancer treatment. With significantly diminished access to their provider post-treatment, survivors lacked a dependable resource to share their concerns, and ask questions. It was observed that while the SCP provided clinical recommendations, it lacked general resources for several basic needs, for instance, recommended retailers that supplied wigs.

### Step 2 – design and development of ACESO

**Fig. 3:**
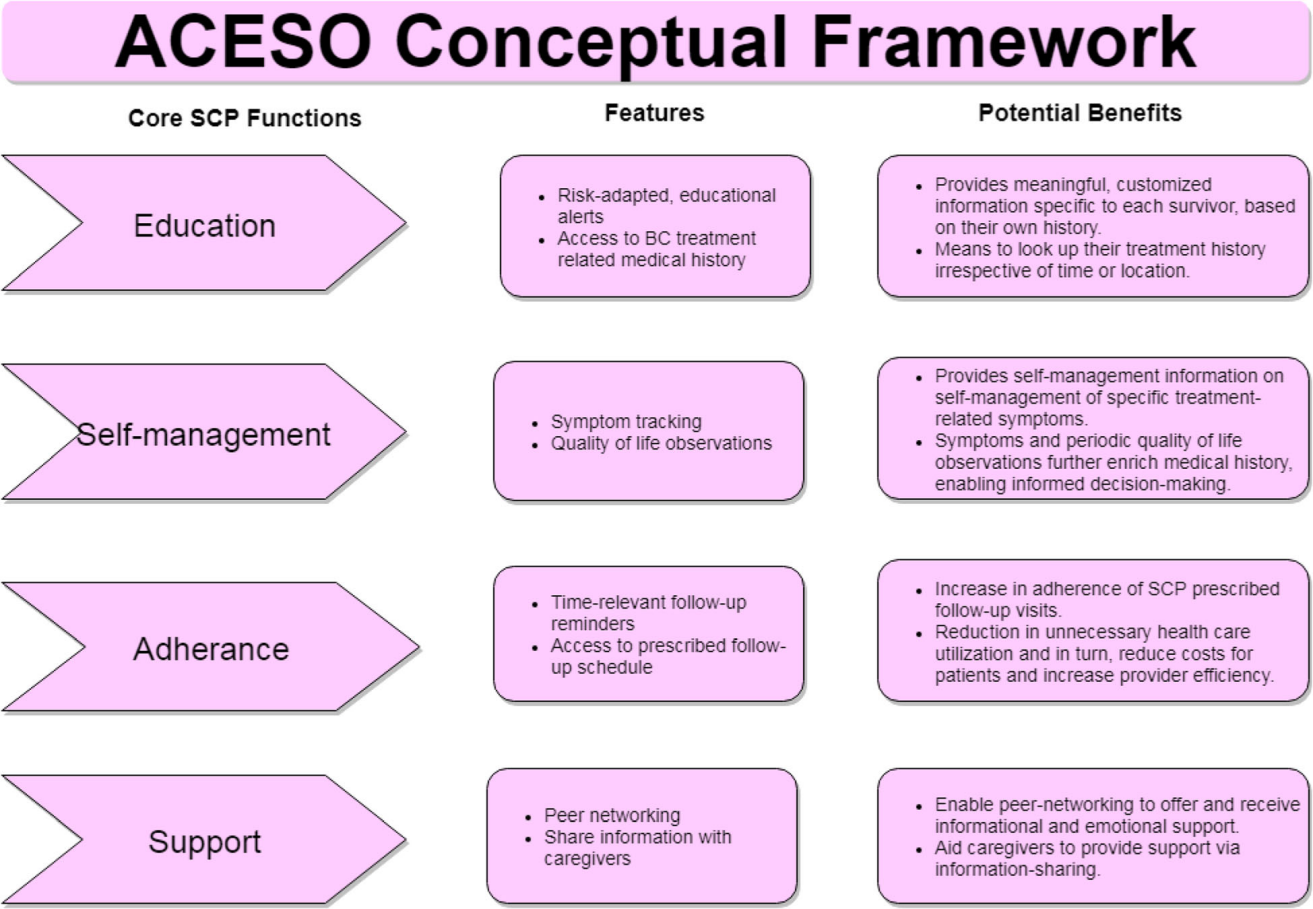
Conceptual framework for implementation of an online SCP, depicting its core functions, features, and benefits *(created with draw.io v. 12.4.2)*

Using the four identified themes in the previous step as proxy for desired core SCP functions, eight Web 2.0 features were identified and matched with them to formulate a conceptual framework for the implementation of the web-based SCP (Fig. 3). The following sub-sections describe the user interface and each of these incorporated features.

#### User interface and features

**Fig. 4:**
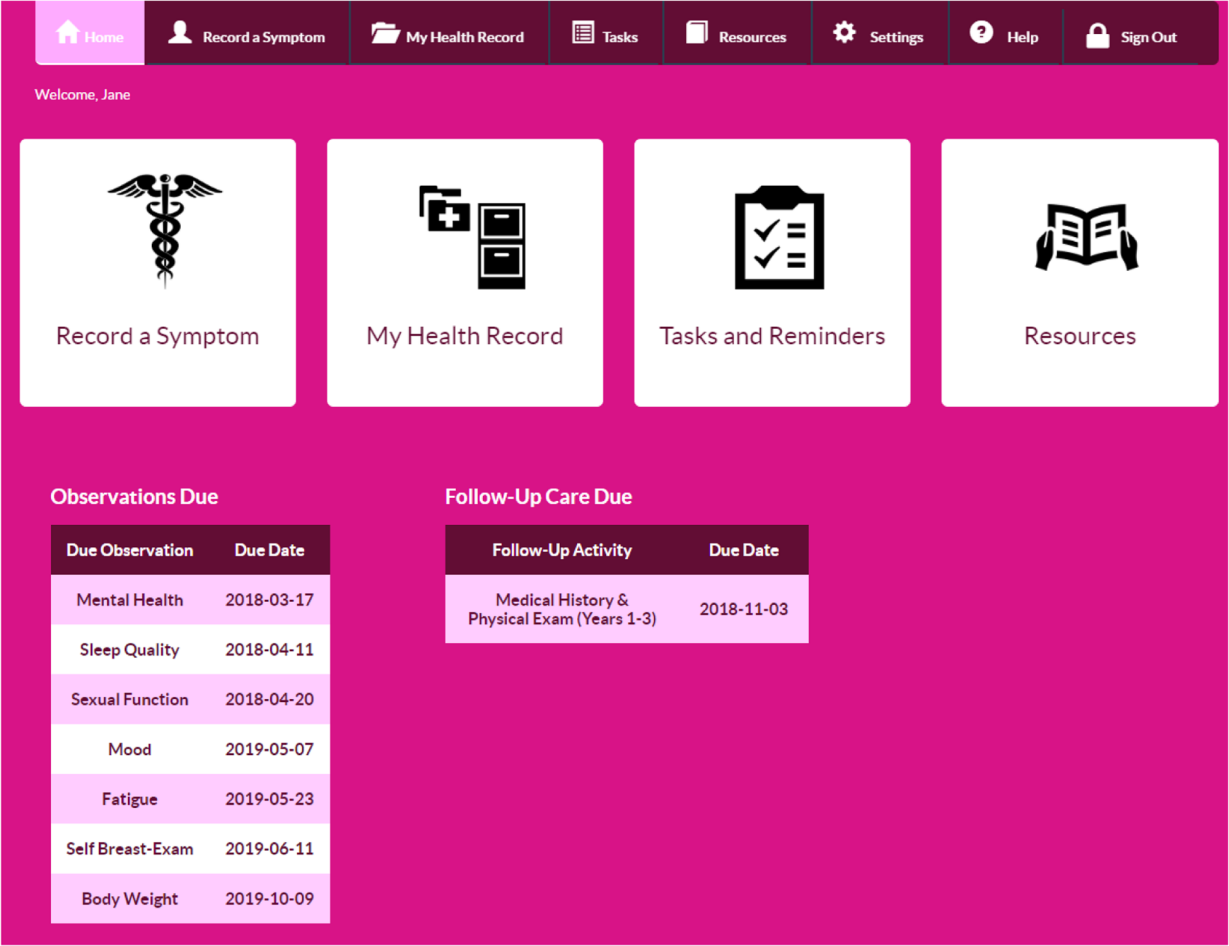
Home screen of the ACESO online SCP tool

When the user logs in to ACESO, they are presented with options to view their breast cancer related medical history, record observed symptoms, or view upcoming reminders for recording home observations or follow-up visits (Fig. 4).

#### Symptom tracker

In order to ease the entry of various BC treatment-related symptoms and reduce the burden of sifting through a long list of symptoms, we categorized common survivor symptoms using an expandable list of three top-level symptom domains: physical; mental and emotional; and sexual function. In addition, if a specific symptom was not provided as an option, the BC survivor may select the ‘Other’ option and report the observed symptom using plain text. While recording any specific symptom, the user also has the option to record the date when they first observed the symptom. This information may be used to record and present a timeline of all symptoms observed at home, in a chronological order, which may be printed and shared with a clinician.

#### Tracking quality of life observations

The primary purpose of this feature is to identify areas of concern affecting the survivor population and provide corresponding self-management recommendations. To capture various quality of life observations, ACESO utilizes previously developed instruments that have demonstrated high internal consistency and validity in prior studies with cancer patients. All observations are self-reported using an online questionnaire at specified intervals. In addition to displaying a list of past due observations, ACESO also reminds survivors with an email each time an observation is due to be recorded. A list of all quality of life measures captured in ACESO and their corresponding instruments and capture intervals are shown in Table 2.

**Table 2:** Quality of life measures captured by ACESO and corresponding measures and capture frequencies

Quality of Life Measure	Measurement Instrument	Frequency
Mood	Clickable emoticons	Daily
Fatigue	Brief Fatigue Inventory [35]	Weekly
Weight	User-owned weighing scale	Weekly
Mental Health	CES-D Scale (short form) [36]	Weekly
Sexual Function	Female Sexual Functioning Index [37]	Weekly
Sleep Quality	Pittsburgh Sleep Quality Index (PSQI) [38]	Monthly

**Fig. 5:**
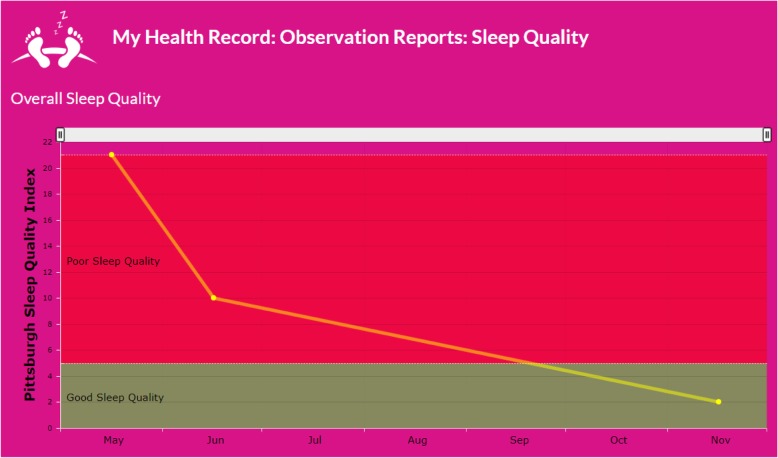
Graphical representation of prior sleep quality observations

Survivors may also view graphical representations (Fig. 5) of previously recorded observations to give them a bigger picture of the overall trend. These records may also be printed and shared with clinicians or caregivers, to identify primary concerns and facilitate informed decision making.

#### Personalized educational alerts

**Fig. 6:**
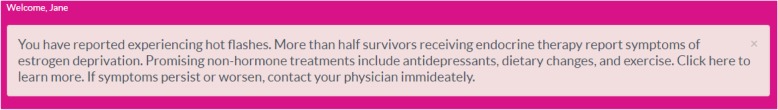
Display of alert message displayed after user indicates upper arm swelling

**Fig. 7:**
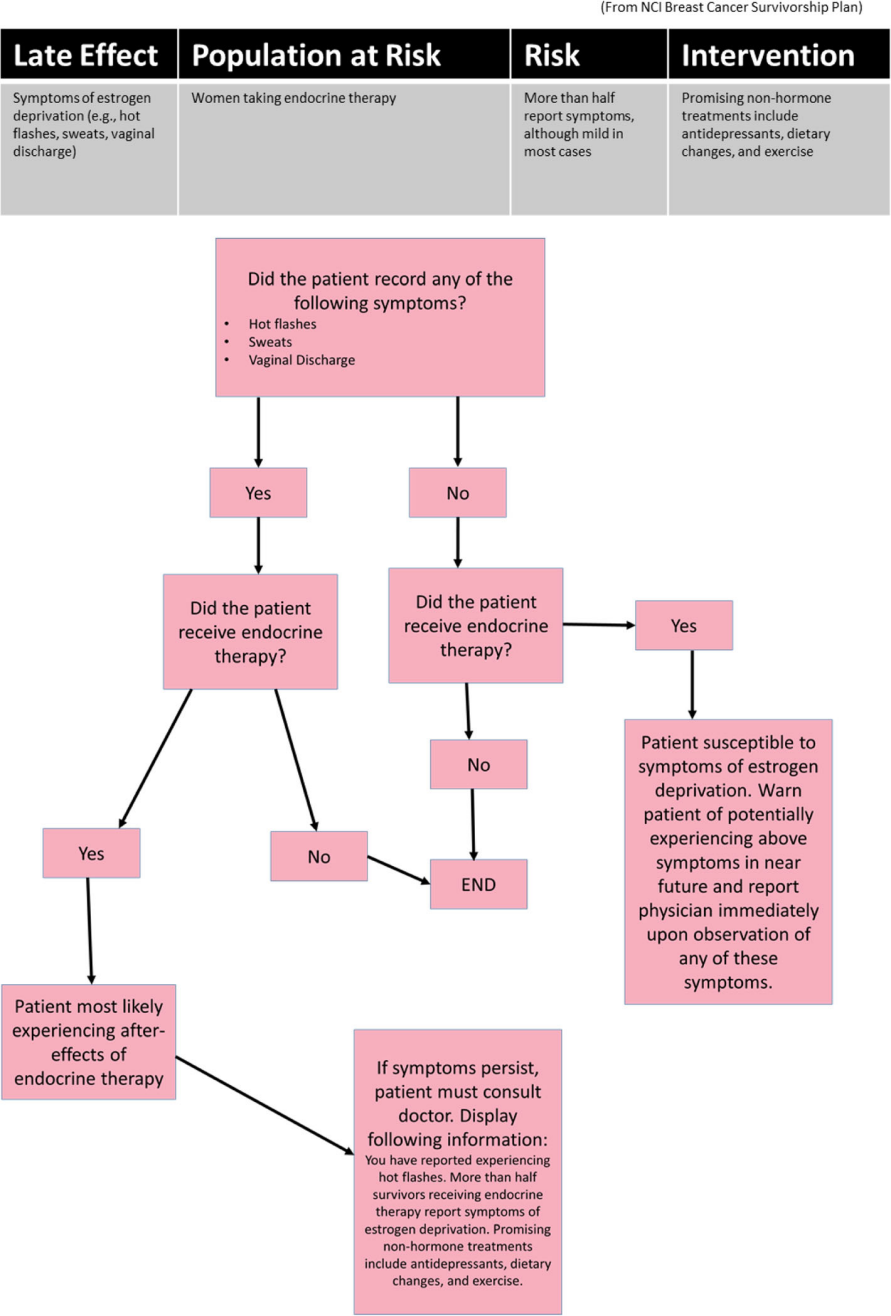
The NCI survivorship care recommendation and the corresponding decision tree for symptoms of estrogen deprivation *(created with draw.io v. 12.4.2)*

ACESO segments the exhaustive amount of textual information in a paper-based survivorship care plan and displays only personalized educational information on self-management and healthy behaviors in the form of alerts and reminders, which are most relevant to the survivor at any given time. These alerts are risk-adaptive (Fig. 6), based on each survivor’s own BC related medical history and quality of life, making them customized and tailored for each user. For instance, a common treatment-related side effect from undergoing endocrine therapy, is the development of menopausal symptoms. Figure 7 illustrates the NCI guideline and the corresponding decision tree representation of the knowledge used in ACESO.

#### Follow-up reminders

While paper-based survivorship care plans summarize and list follow-up care for each patient, its passive nature does not allow for reminding the patient at the right time. This conventional form of a survivorship care plan relies on the patient to recall their follow-up schedule over the several years of survivorship. This is potentially problematic, especially since certain follow-up visits, such as bone densitometry and pelvic examinations do not occur as frequently as post-treatment mammography visits, or even annual comprehensive check-ups. ACESO attempts to reduce this burden on BC survivors by only displaying upcoming follow-up visits, or those that are past due in the form of a to-do list. If a BC survivor does not log in to ACESO frequently, an email reminder is also sent to the patient 2 weeks prior to each expected follow-up visit, to remind them and allow time to plan, and schedule an appointment with their health care provider.

#### BC treatment related medical history

This section of ACESO lists specific components of each BC survivor’s treatment related medical history. This information is derived from the original paper-based survivorship care plan document and entered into ACESO by each user at the time of sign-up and registration. Having an electronic record of their treatment history, provides a more portable version of the paper-based survivorship care plan, which may be accessed online irrespective of time and location. This feature provides a means for the patient to potentially share their information with caregivers, or other providers.

#### Peer networking

ACESO provides a platform to enable online discussion and communication among BC survivors. The discussion board allows survivors, especially those living in remote areas to share their experiences of BC treatment, in addition to provide emotional and informational support to each other, including alternative therapies, provider recommendations and strategies for long-term survival.

### Step 3 – feasibility evaluation

One hundred thirty BC survivors who met screening requirements registered and created personal accounts on ACESO. Eighty nine of these registered users (68%) logged into ACESO more than once, after creating their account the first time. Of all the 130 registered users, 51 completed the survey at the end of 6 weeks (Table 3). Participants were asked to provide their preliminary perception of their use of ACESO.

**Table 3:** Demographic characteristics of the participants in the feasibility test (*n* = 51)

	Frequency (n)	(%)
**Age**		
18–39	28	55
40–49	17	33
50 and above	6	12
**Race**		
Caucasian	41	80
Hispanic or Latino	4	8
Other	6	12
**Education (highest level completed)**		
Associates/Technical degree	7	14
Bachelor’s degree	29	57
Master’s degree	12	23
Doctoral degree	3	6
**Marital status**		
Married	48	94
Divorced or separated	3	6
**Employment Status**		
Employed full-time	40	78
Employed part-time	6	12
Other	5	10

Overall, participants expressed high levels of satisfaction from using ACESO and indicated that it was a useful tool for BC survivors. Descriptive statistics describing user feedback is provided in Table 4.

**Table 4:** Survivors’ feedback on the online SCP after 6 weeks of use

Question	Yes	No
Do you think ACESO is a useful tool for breast cancer survivors?	46 (96%)	2 (4%)
Would you recommend ACESO to other breast cancer survivors?	48 (94%)	3 (6%)
Overall, did ACESO have an impact in self-managing and/or improving your quality of life?	47 (92%)	4 (8%)

The survey also asked open-ended questions for the survivors to provide their initial thoughts and feedback on ACESO. In terms of what they liked about ACESO, participants mentioned “*everyday reminders*”, “*building confidence*”, “*simplicity*”, and “*the self-check*”. One respondent stated “*It reminded me to take a few minutes to reflect on how I was feeling and document it. I have been having a lot of pain since my last surgery, so it was good to document that and evaluate my next steps for treatment*”. Another participant commented that “*The resources are abundant*”.

Participants also provided feedback on areas for improvement. Some comments indicated the need for a companion mobile app, and that the “*terminology is too professional*”. The tool utilizes the Female Sexual Functioning Index (FSFI) [37] to measure sexual function, however it was not accommodating of women who were single. As one user stated that “*the sexual health survey and automated response assumed the individual is in a relationship*”. In total, twenty participants indicated that they would not change anything in the current prototype.

## Discussion

As BC survivors transition from seeing oncologists to primary care, they may have to assume the role of messenger between the two providers, and fill in the communication gaps, which is instrumental in promoting continuity of care [39]. Survivors suddenly find themselves having to take care of themselves, often without the proper training and understanding of their current condition, and what to expect in the near future, in the form of side effects of treatment as well as possible recurrence [40]. They face several barriers, such as the lack of knowledge and understanding of their medical condition, coupled with the lack of specific tools and resources that enable them to achieve this [40, 41]. This need for personalized and customized education pertaining to their BC treatment was consistent with our findings.

Survivors expressed their desire to play a more active role in managing their health post treatment. This finding is consistent with prior evidence indicating survivors’ desire to play a more active role in managing their health conditions [12, 42]. ACESO incorporates real-time feedback, coupled with self-regulatory tools, such as symptom tracking, and the logging and charting of quality of life observations; features absent on conventional survivorship care planning.

While the conventional SCPs provide a schedule of recommended follow-up visits, they currently lack the ability to remind survivors of upcoming visits, which can be a barrier, especially several years after treatment. Both under-utilization and over-utilization of health care services has been shown to be a problem among survivors [43], therefore it is hoped that the reminder function could potentially mitigate this issue. Providing timely email reminders for follow-up should also improve adherence for post-treatment clinical care.

Furthermore, ACESO provides a platform for survivors to connect with peers and provide emotional support to each other post-treatment. In addition to providing emotional support, peer-networking can connect survivors, enabling them to share such supportive information regarding basic needs [44, 45].

ACESO serves as an organic and living SCP, and dynamically adapts to their needs over the long period of time and presents only relevant alerts and self-management education for their most current needs. In the context of chronic care, the modelling of clinical survivorship guidelines into a connected system intended for continued and long-term use allows for rapid updates as new guidelines are released. This ensures the delivery of the most recent and evidence-based recommendations to survivors, in comparison to providing a static paper document once, with gradually extant pieces of information.

It is hoped that this tool will empower survivors, enabling them to take charge of their health on their own hands, participate in shared decision-making, and ask better, informed questions from their provider.

### Limitations

As a completely patient-managed system, ACESO relies on the individual survivor’s skills and competence to ensure accuracy of the entered data. To mitigate errors in data entry, as part of future updates, ACESO may allow for automated capture of certain observations, such as sleep quality by exploiting sensors and accelerometers found in consumer mobile devices. ACESO is not intended to provide clinical advice or perform clinical diagnoses, rather as an educational tool for survivors to aid self-management. Consequently, all users are informed during the sign-up process that the tool does not replace a medical professional and they must always seek professional help of a clinician when needed. To further re-enforce this message, the text of all alerts contains information to contact a physician if their condition does not improve or worsens.

### Future steps

More exhaustive evaluation is necessary to evaluate ACESO’s ease of use and its overall effectiveness in improving the survivors’ knowledge, self-efficacy, adherence to follow-up, and perceived social support. As a completely patient-managed system, ACESO relies on the individual survivor’s skills and competence to ensure accuracy of the entered data. To mitigate errors in data entry, as part of future updates, a mobile app version of ACESO may allow for automated capture of certain observations, such as sleep quality, by exploiting sensors and accelerometers found in consumer mobile devices. Alternative instruments to measure overall female sexual health, rather than sexual function should be incorporated to make the application more inclusive for all women.

## Conclusion

As a one-of-a-kind educational and personal decision support tool, ACESO has the potential to significantly improve the standard discharge procedure of BC survivors, and attempts to address the several shortcomings of a conventional paper-based survivorship care plan. Rather than providing exhaustive information to survivors at the time of discharge, ACESO provides risk-adapted alerts and time-relevant reminders at the point of need, rather than point of care. In contrast with a paper-based SCP that provides extensive amounts of advisory information all at once, thereby overwhelming the patient, ACESO segments the pieces of information most relevant for the survivor at any given time and presents it to them in an interactive format, which is known to aid active learning and enhance cognition [46]. The framework presented in this study can be adopted for the implementation of personal decision support utilizing survivorship guidelines for other forms of cancer, or other chronic conditions.

## Additional file

**Additional file 1.** Talking points to assess survivor needs and preferences during Step 1 of the study

## Data Availability

The datasets generated and/or analyzed during the current study are not publicly available due to individual privacy could be compromised but may be available from the corresponding author on reasonable request.

## Abbreviations

ACESO: After cancer education and support operations
ASCO: American Society for Clinical Oncology
BC: Breast cancer
CES-D: Center for epidemiologic studies – depression scale
FSFI: Female sexual functioning index
IRB: Institutional review board
NCI: National Cancer Institute
PGHD: Patient generated health data
PHP: PHP hypertext preprocessor
PSQI: Pittsburgh sleep quality index
SCP: Survivorship care plan
SNOMED-CT: Systemized nomenclature of medicine – clinical terms
