# Supplementary material for: Personal decision support for survivor engagement: formulation and feasibility evaluation of a conceptual framework for implementing online cancer survivorship care plans
Source: BMC Med Inform Decis Mak. 2020 Mar 23;20:59. doi: 10.1186/s12911-020-1073-8 (PMC7092430; doi:10.1186/s12911-020-1073-8)
Supplement: Supplementary file 1 — Additional file 1. Talking points to assess survivor needs and preferences during Step 1 of the study [file 12911_2020_1073_MOESM1_ESM.docx]

| Q1 | After completing your cancer treatment, how well prepared did you feel in terms of taking care of yourself and follow up treatments? |
| --- | --- |
| Q2 | What were the major challenges which you faced after completing your cancer treatment? |
| Q3 | How useful did you find the breast cancer survivorship document given to you by your provider after you completed your cancer treatment? |
| Q4 | How do you feel about using technologies, such as mobile apps to manage your health condition? |
| Q5 | If you were to design an app for breast cancer survivors, what features would you like to see in such an app? What would it look like? |
| Q6 | Do you have any concerns from using such an app? If yes, what are they? |
| Q7 | Any other comments for me? |

Talking points to assess survivor needs and preferences during Step 1 of the study
